# Metabolite analysis of tubers and leaves of two potato cultivars and their grafts

**DOI:** 10.1371/journal.pone.0250858

**Published:** 2021-05-06

**Authors:** Khongorzul Odgerel, Zsófia Bánfalvi

**Affiliations:** NARIC, Agricultural Biotechnology Institute, Gödöllő, Hungary; Huazhong Agriculture University, CHINA

## Abstract

Grafting experiments have shown that photoperiod-dependent induction of tuberisation in potato (*Solanum tuberosu*m L.) is controlled by multiple overlapping signals, including mobile proteins, mRNAs, miRNAs and phytohormones. The effect of vegetative organs and tubers at metabolite level and vice versa, however, has not been studied in detail in potato. To unravel the influence of vegetative organs on the primary polar metabolite content of potato tubers and the effect of tuberisation on the metabolite content of leaves grafting experiments were carried out. Two potato cultivars, Hópehely (HP) and White Lady (WL), were homo- and hetero-grafted, and the effects of grafting were investigated in comparison to non-grafted controls. Non-targeted metabolite analysis using gas chromatography-mass spectrometry showed that the major difference between HP and WL tubers is in sucrose concentration. The sucrose level was higher in HP than in WL tubers and was not changed by grafting, suggesting that the sucrose concentration of tubers is genetically determined. The galactinol level was 8-fold higher in the WL leaves than in the HP leaves and, unlike the sucrose concentration of tubers, was altered by grafting. A positive correlation between the growth rate of the leaves and the time of tuber initiation was detected. The time of tuber initiation was delayed in the WL rootstocks by HP scions and shortened in the HP rootstocks by WL scions, supporting the previous finding that tuberisation is triggered by source-derived mobile signals.

## Introduction

Potato tuber is an underground vegetative storage organ differentiated from stolons. In strict short-day tuberising potato genotypes, such as *Solanum tuberosum* L. subsp. *andigena*, the tuberisation is tightly linked to photoperiod. Grafting experiments have proven that the initiation of tuberisation is mediated by graft-transmissible signals, such as mobile proteins, mRNAs, miRNAs and phytohormones [1].

The photoreceptor PHYTOCHROME B (PHYB) has been shown to be involved in the response of potato tuber induction to the photoperiod. Using an antisense approach, Jackson et al. [2] showed that *PHYB*-repressed *S*. *andigena* plants tuberise during long days, and the effect was found to be graft-transmissible. Grafting experiments performed by Martínez-García et al. [3] suggested the existence of two independent pathways that control tuber formation: a photoperiod-dependent pathway and a gibberellic acid (GA)-dependent pathway. Grafting of photomorphogenic and GA-deficient or hypersensitive tomato mutants onto potato rootstocks supported this finding [4]. Chailakhyan et al. showed as early as 1981 [5] that grafting flowering tobacco shoots into potato rootstocks promoted tuberisation in the rootstocks, indicating that the floral- and tuber-inducing signals might be similar. Later, evidence was obtained for a conserved function of the potato orthologues of the CONSTANS (CO) and FLOWERING LOCUS T (FT)-like transcription factors SP3D, SP6A, TFL1 and SP5G in tuberisation control during short days [6–8]. In addition, two transcription factors, the BEL1-like BEL5 and KNOTTED-TYPE HOMEOBOX (KNOX), designated POTH1 (POTATO HOMEODOMAIN 1) in potato, were shown to interact in a tandem complex and regulate tuber formation [9]. Both *BEL5* and *KNOX1* function as phloem mobile mRNAs, which move from leaf to stolon [10–12]. CO has been found to repress tuberisation in a photoperiod-dependent manner, and this effect was transmitted through grafts [13]. Kloosterman et al. [14] identified a central regulator for the initiation of tuber development. This gene, *CYCLING DOF FACTOR 1* (*CDF1*), regulates tuberisation by acting as a mediator between the circadian clock and the mobile SP6A peptide tuberisation signal.

The roles of miRNAs in tuberisation were first demonstrated by Martin et al. [15], who showed that overexpression of *miR172* in potato triggered tuber formation during long days. Bhogale et al. [16] demonstrated that another miRNA, miR156, is a graft-transmissible signal that affects plant architecture and tuberisation in potato by regulating miR172 in *S*. *tuberosum* subsp. *andigena*.

In addition to the above-described transcription factors and miRNAs, various other proteins have been reported to be associated with tuberisation in potato [17]. Nevertheless, at least to our knowledge, no reports on the tuberisation and metabolite composition of leaves and tubers of grafted potato cultivars are available in the literature. There are several publications on tuber metabolome ([18] and references therein) since the pioneer work of Roessner et al. [19] and also on leaf metabolome mainly in relation to different stress responses [20, 21] and genetic modifications [22, 23]. However, the effect of leaves on metabolite composition of tubers and vice versa has not been studied in detail in potato. Thus, the aim of our work was to study these effects by grafting experiments using two commercial potato cultivars, Hópehely (HP) and White Lady (WL) with contrasting tuber metabolite compositions [24]. Here, we report that hetero-grafting affected the canopy and tuber development of plants and slightly influenced the galactinol concentrations in leaves. However, it had no significant influence on the metabolite composition of tubers, including sucrose, the major polar metabolite in tubers.

## Materials and methods

### Plant materials and growth conditions

Two potato cultivars, *Solanum tuberosum* cv. Hópehely (HP) and White Lady (WL) originating from the breeding programme of the Potato Research Centre, Keszthely, Hungary, were propagated in RM culture medium (MS medium without vitamins) [25] supplemented with 2% (w/v) sucrose and 0.8% agar. In total, 25–30 plantlets from each cultivar were grown in test tubes at 24°C under a photoperiod cycle of 16 h/8 h day/night at a light intensity 75 μmol m^-2^ s^-1^. After four weeks of culturing, *in vitro* plantlets with 3–4 cm long roots were transferred into polyethylene pots (8 cm diameter top and 6 cm depth) containing Tabaksubstrat sterile soil A200 (Stender GmbH, Schermbeck, Germany) and placed into a plastic box with a lid. The plantlets were acclimatised for two weeks under ambient light conditions supplemented with artificial lightening by sodium lamps (12 h day/12 h day/night cycle) in a greenhouse at a temperature regime of 18–24°C and soil humidity up to 80% provided by regular watering. After a successful acclimatisation period, the plants were used for grafting experiments.

### Grafting and plant phenotyping

Three consecutive grafting experiments were carried out. For each experiment, 15–20 plants of the two potato cultivars were grafted by the splice grafting method. Stock plants were decapitated 2–3 cm above the root shoot joint and served as rootstocks without leaves. Scions were prepared by cutting the plants with a diagonal cut through the internodal part of the stem at the same distance above the ground as for the rootstocks. The scions were fitted to the stock and wrapped with rubber clips. In all the experiments, three groups of the two cultivars (non-grafted control and homo-grafted and hetero-grafted HP and WL) were used. For four days after grafting, the plants were incubated in a plastic box with a lid under high humidity in darkness at 24°C. The non-grafted and grafted plants were grown further under greenhouse conditions (see above) for one week and then transferred to pots with a 14 cm diameter top and 14 cm depth. Six weeks after grafting, the plants, together with the soil, were carefully tipped out of the pots, checked for tuber formation and re-planted. The five largest leaves from each plant were harvested, and their area was measured to estimate canopy development. At the end of the vegetation period, the tubers were harvested, visually evaluated for shape and colour and measured to obtain fresh weight yield.

### Metabolite extraction and profiling

Six weeks after grafting, three to four sets of leaves, each containing three leaf disks 1 cm in diameter originating from three individual plants, were prepared, frozen in liquid nitrogen and stored at -70°C until they were used for metabolite extraction. After harvesting, four to five sets of tubers, each containing three tubers approximately 1.5–2.0 cm in diameter, were prepared and washed well with deionised water. Approximately 0.1–0.2 cm-thick freshly cut radial slices taken from the centres of the tubers were cut into small pieces, frozen in liquid nitrogen and stored at -70°C. Both the leaf and tuber samples were ground into fine powder in a mortar with a pestle. Metabolite extraction and profiling were performed as described in detail by Nikiforova et al. [26]. Ribitol (0.2 mg ml^-1^) was added to the extract as an internal standard. N-methyl-N-(trimethylsilyl) trifluoroacetamide (MSTFA) was used for derivatisation, and the samples were analysed in a quadrupole-type gas chromatography-mass spectrometry (GC-MS) system (Finnigan Trace/DSQ, Thermo Electron Corp., Austin, TX, USA) equipped with a 30 m capillary column (Rxi-5 ms, 0.25 mm ID, 0.25 μm df, Restek) in TIC (total ion chromatogram) mode. Mass spectra were recorded at 0.8170 scans/sec with a m/z 50–650 scanning range. Thermo Scientific Xcalibur software was used for exporting the spectra and searching the NIST 11 mass spectral database. In addition, the sugars were identified based on a comparison of the retention time and mass spectrum to an authentic standard that was analysed under identical conditions. The raw data are available in S1 and S2 Tables.

### Statistical analysis

Quantitative phytochemical analysis was performed by principal component analysis (PCA) and variable importance projection (VIP) plots by partial least squares discriminant analysis (PLS-DA) of the first principle components (MetaboAnalyst 4.0, https://www.metaboanalyst.ca). A value of 1.0 was selected as the cut-off for the VIP values. As pre-treatment, sample normalisation was carried out based on median values, and the data were log transformed. Significant differences in galactinol and sucrose contents were detected by one-way ANOVA with a post hoc Tukey HSD test.

## Results

### Effect of grafting on growth and tuberisation

**Fig 1:**
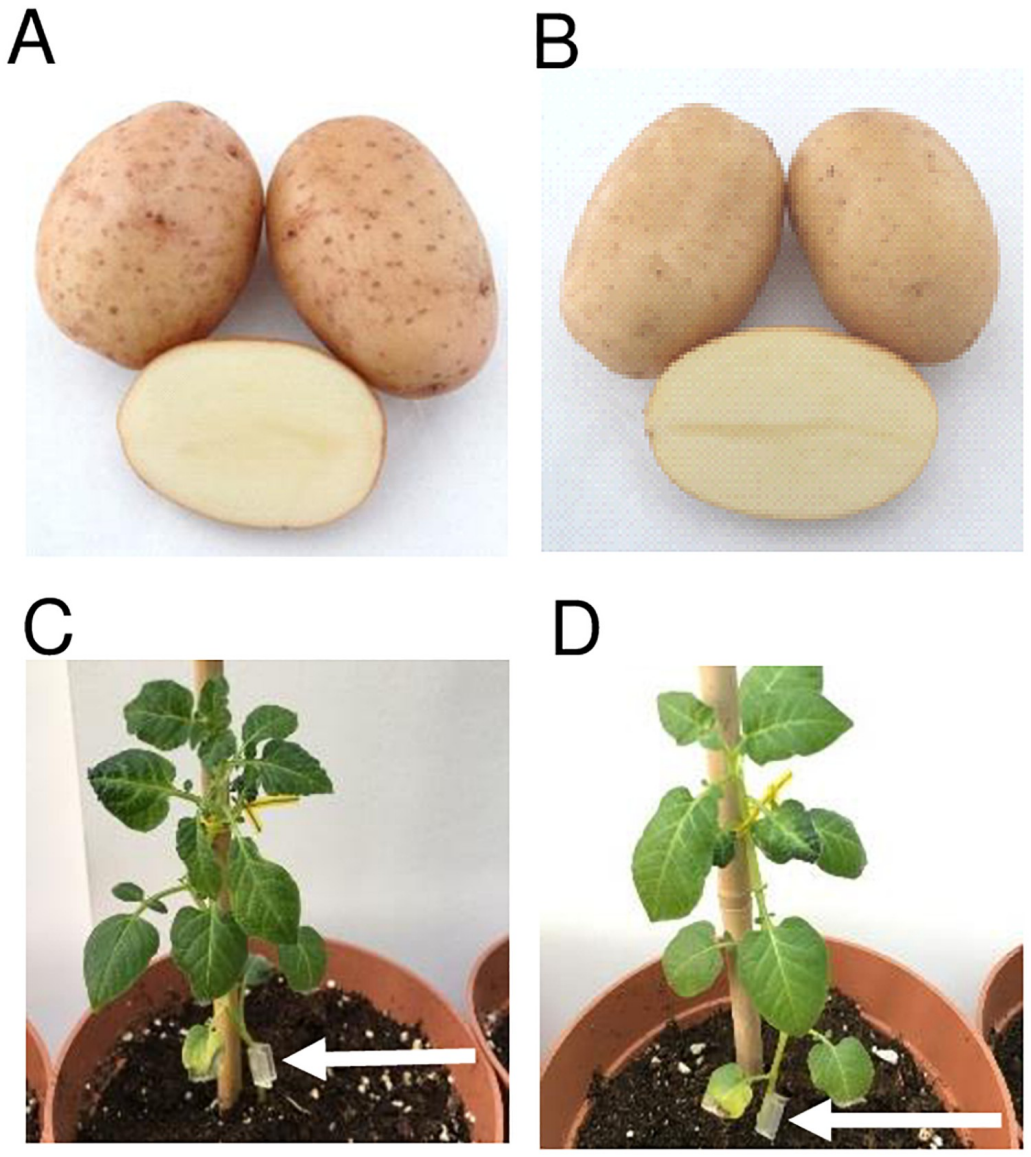
Tuber and canopy morphology of two Hungarian potato cultivars, Hópehely (A and C) and White Lady (B and D). Tubers were grown in field conditions (photography by Z. Polgár). The canopy morphology of the greenhouse-grown plants is shown. Four-week-old *in vitro* plants were potted and used for grafting experiments two weeks after potting. The arrows indicate the scion/stock junctions.

**Fig 2:**
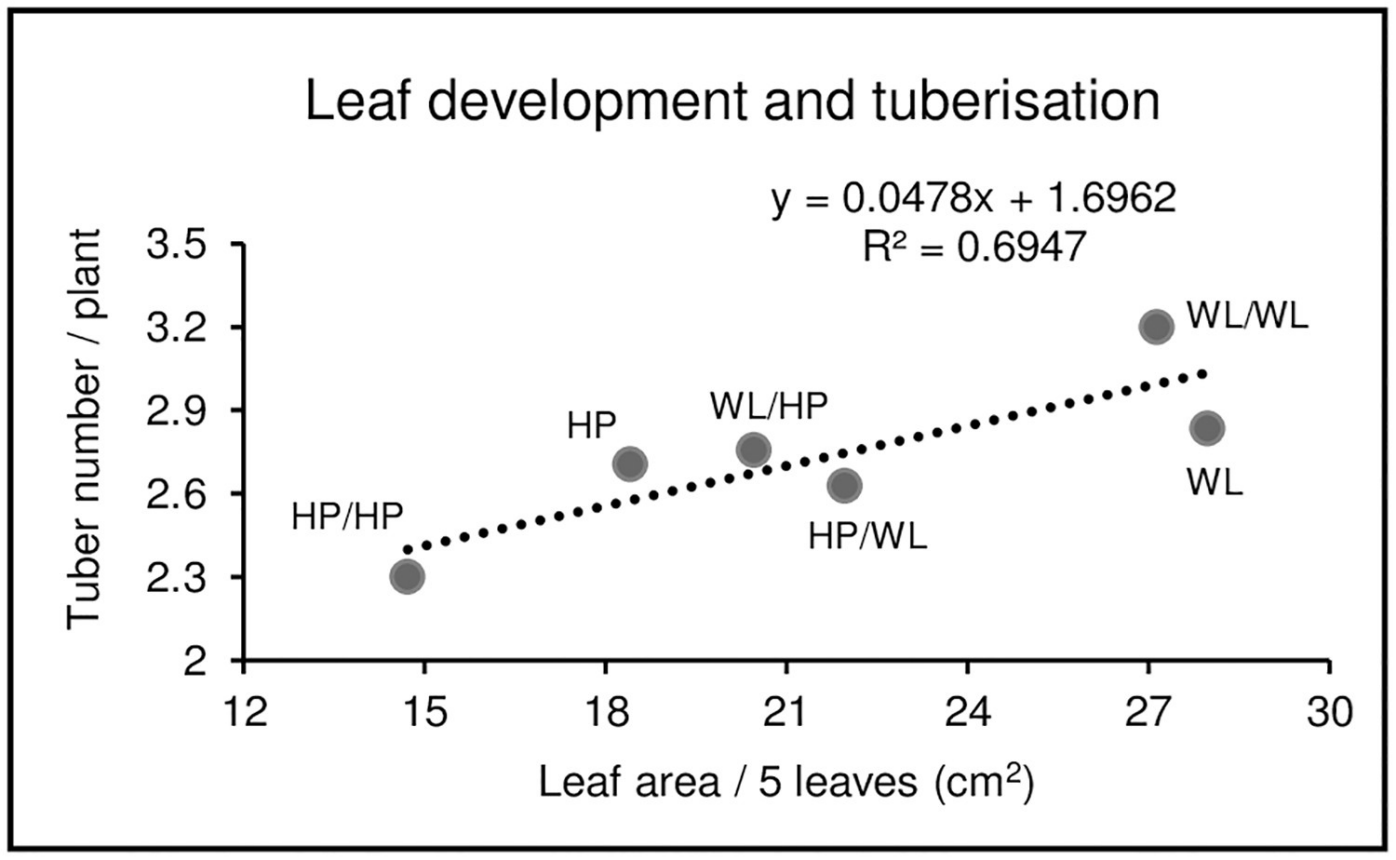
Correlation between leaf size and tuberisation six weeks after grafting. The area of the five largest leaves per plant was measured. Three consecutive experiments were carried out. In sum, 20–25 plants were tested in each experiment. HP, non-grafted control Hópehely; WL, non-grafted control White Lady; HP/HP, homo-grafted Hópehely; WL/WL, homo-grafted White Lady; HP/WL, hetero-graft: Hópehely scion/White Lady rootstock; WL/HP, hetero-graft: White Lady scion/Hópehely rootstock.

To investigate the influence of grafting on the growth and tuberisation of the potato cultivars Hópehely (HP) and White Lady (WL), which have different tuber morphologies (Fig 1A and 1B), plantlets grown *in vitro* were transferred into pots and grown further under greenhouse conditions. After two weeks of acclimatisation, homo- and hetero-grafts were prepared and grown further, together with non-grafted control plants, in a greenhouse. Two weeks after grafting, plants with well-developed root and shoot systems (Fig 1C and 1D) were counted. Three consecutive experiments were carried out, with a grafting success of 64–85%. Six weeks after grafting, the number of tubers was tested by tipping the plants out of the pots. The canopy development of the plants was also estimated at this time by measuring the area of the five largest leaves of the plants. A positive correlation (*R*^*2*^ = 0.6947) between the leaf growth rate and the number of tubers formed on plants was detected (Fig 2). The leaf area as well as the number of tubers was low in the case of HP and homo-grafted HP/HP plants, while these parameters were high in the case of WL and WL/WL plants. The hetero-grafts, either WL (HP/WL) or HP (WL/HP) of the rootstock, possessed intermediate values, indicating that canopy development and tuber initiation are influenced by grafting.

At the end of the vegetation period, the mature tubers were collected and visually characterised, the number of tubers per plant was counted and the mass of the tubers was measured. The HP tubers were roundish with yellow to pinkish skin colour (S1A Fig), while the WL tubers were short-oval and white-yellowish (S1B Fig). The grafting did not change the colour of the tubers (S1C–S1F Fig). In contrast, the shape of several tubers collected from hetero-grafted plants resembled that of the scion tubers (S1A, S1B, S1E and S1F Fig). The average number of tubers per pot ranged from 2.3 to 3.2 (S2A Fig), with a mass of 2.6 to 3.6 g/tuber (S2B Fig). The tuber yield was between 7.2 and 10.2 g/plant (S3C Fig). There was no significant difference in these parameters between the HP and WL plants, and the values were not changed by grafting.

### Metabolic profiling of leaves

To investigate the influence of grafting on the metabolite composition of leaves, samples were collected six weeks after grafting in two consecutive experiments, and metabolite profiling was carried out using GC-MS. A total of 31 polar metabolites were identified in the leaf extracts (S1 Table), including 15 different amino acids, 4 sugars, 3 sugar alcohols, 8 organic acids and 1 inorganic acid (S3 Fig). The major compounds in the leaves were sugars and malic acid. The concentrations of sugars in the leaves were quantified by comparing the peak sizes of the leaf samples to those of authentic standards. Glucose, fructose and sucrose were present in the highest amounts at concentrations of 1.13±0.26, 1.10±0.27 and 3.52±0.38 mg/g fresh weight (FW) in the HP leaves, while the concentrations of the same compounds were 2.15±0.05, 2.57±0.10 and 6.82±0.70 mg/g FW in the WL leaves, respectively. Despite the differences detected in the fructose, glucose and sucrose concentrations in the leaves of the two cultivars, no significant differences were found in the sugar content of the leaves of the hetero-grafted plants compared to their homo-grafted counterparts (S4A Fig), indicating that the sugar content of scions is not influenced by rootstocks. The average concentration of malic acid was very much the same in the two cultivars. However, it fluctuated substantially in the individual groups of samples. Among the amino acids, proline, glutamine (measured as oxo-proline [27]), glutamic acid and aspartic acid had the highest peaks. The main fatty acids were palmitic and stearic acid (S3 Fig).

**Fig 3:**
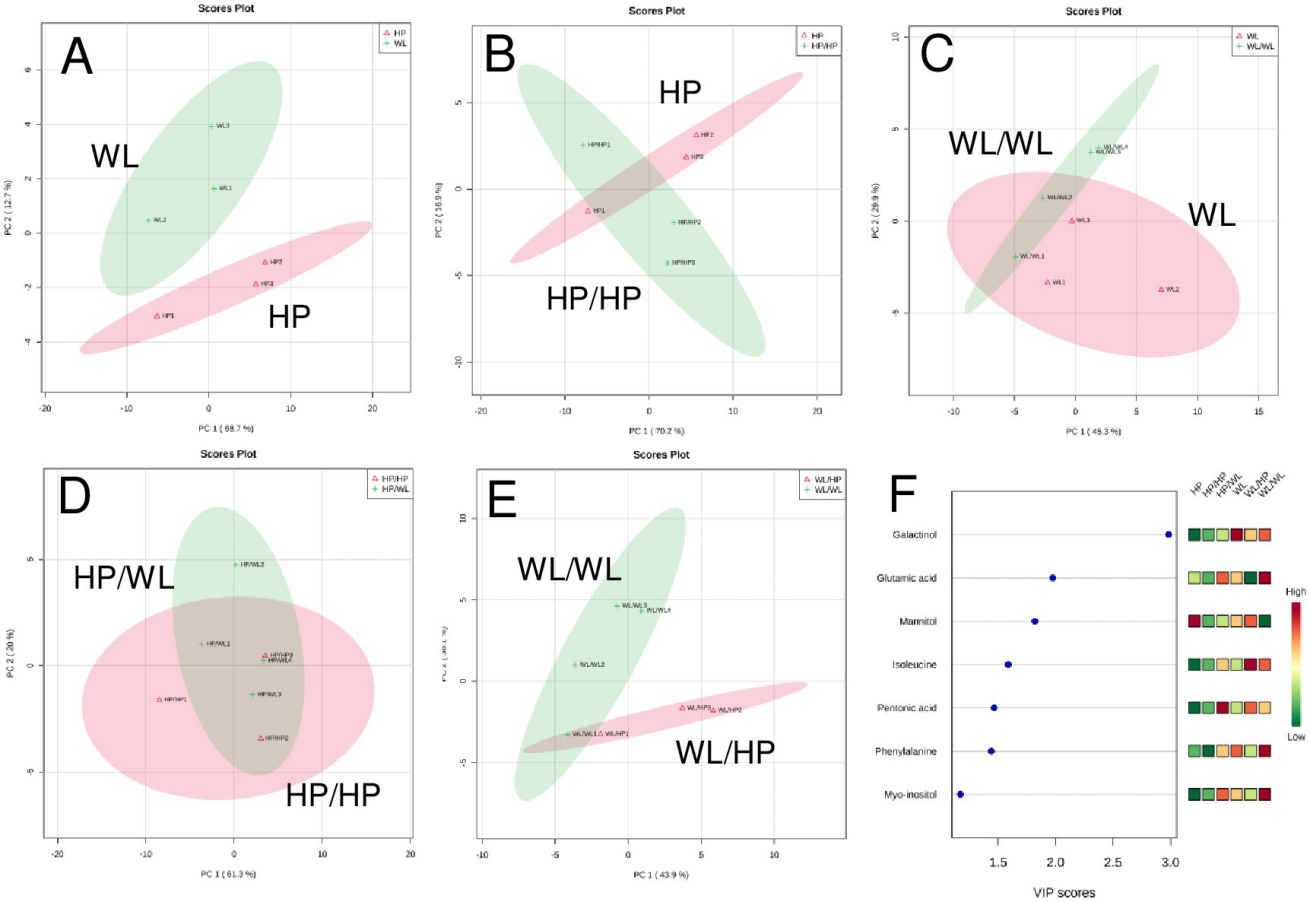
PCA and VIP plots from PLS-DA showing the metabolite differences in the source leaves of non-grafted (A), homo-grafted (B and C) and hetero-grafted (D and E) plants. The VIP plots (F) indicate the major differences between each category. In each plot, the 95% confidence regions are displayed. The leaves were collected six weeks after grafting at four hours after sunrise. The data were obtained from two consecutive experiments with 3–4 groups of samples. Each group contained 3 leaf disks 1 cm in diameter cut from the middle of assimilating leaves of 3 individual plants. The labels are as in Fig 2.

Principal component analysis (PCA) of the HP and WL leaf metabolite data revealed distinct profiles causing samples to cluster based on genotype. The first and second principal components, representing 68.7% and 12.7% of the total sample variance, respectively, clearly separated the HP and WL samples in the loading plots (Fig 3A), indicating that a range of compounds, including galactinol, mannitol, glutamic acid, citric acid and proline, were driving the sample differences (S5A Fig).

Metabolite analysis of the homo-grafted plants, carried out in the same way as for the non-grafted controls, showed that although there were some differences compared to the non-grafted controls (S5B and S5C Fig), interruption of plant development by cutting and healing did not substantially influence the constitution of the leaf metabolome as a whole (S3B and S3C Fig). The hetero-grafting did not substantially change the metabolome of the leaves (Fig 3D and 3E). Nevertheless, compared to the leaves of the homo-grafted plants, the hetero-rootstocks evoked some differences in the leaves, especially in the concentrations of glutamic acid, citric acid, proline, β-alanine and ornithine (S5D and S5E Fig).

To determine which compounds caused the major differences between the leaves of the six sets of plants, variable importance projection (VIP) plots by partial least squares-discriminant analysis (PLS-DA) were utilised. Seven compounds had a VIP score higher than 1.0, out of which four compounds (galactinol, glutamic acid, mannitol and isoleucine) possessed values above 1.5 (Fig 3F).

### Metabolic profiling of tubers

To analyse the metabolite composition of the tubers, three freshly collected mature tubers approximately 1.5–2.0 cm in diameter were grouped together. For tubers derived from each category of plants, four to five groups of tubers were prepared, and the levels of the same 31 metabolites detected in the leaves were tested in the tubers using GC-MS (S2 Table). In the tubers, as in the leaves, sugars were the most abundant metabolites. However, while in the leaves, the sucrose concentration was approximately the same as the concentrations of fructose and glucose (S4A Fig), the sucrose concentration in the tubers was much higher than the concentrations of the other two sugars (S4B Fig). Unlike in the leaves, in which malic acid was the most abundant organic acid, citric acid was the dominant organic acid in the tubers. In terms of amino acids, asparagine was present in the highest amount in the tubers. The fatty acids palmitic and stearic acid were identified in low amounts similar to those detected in the leaves (S6 Fig).

**Fig 4:**
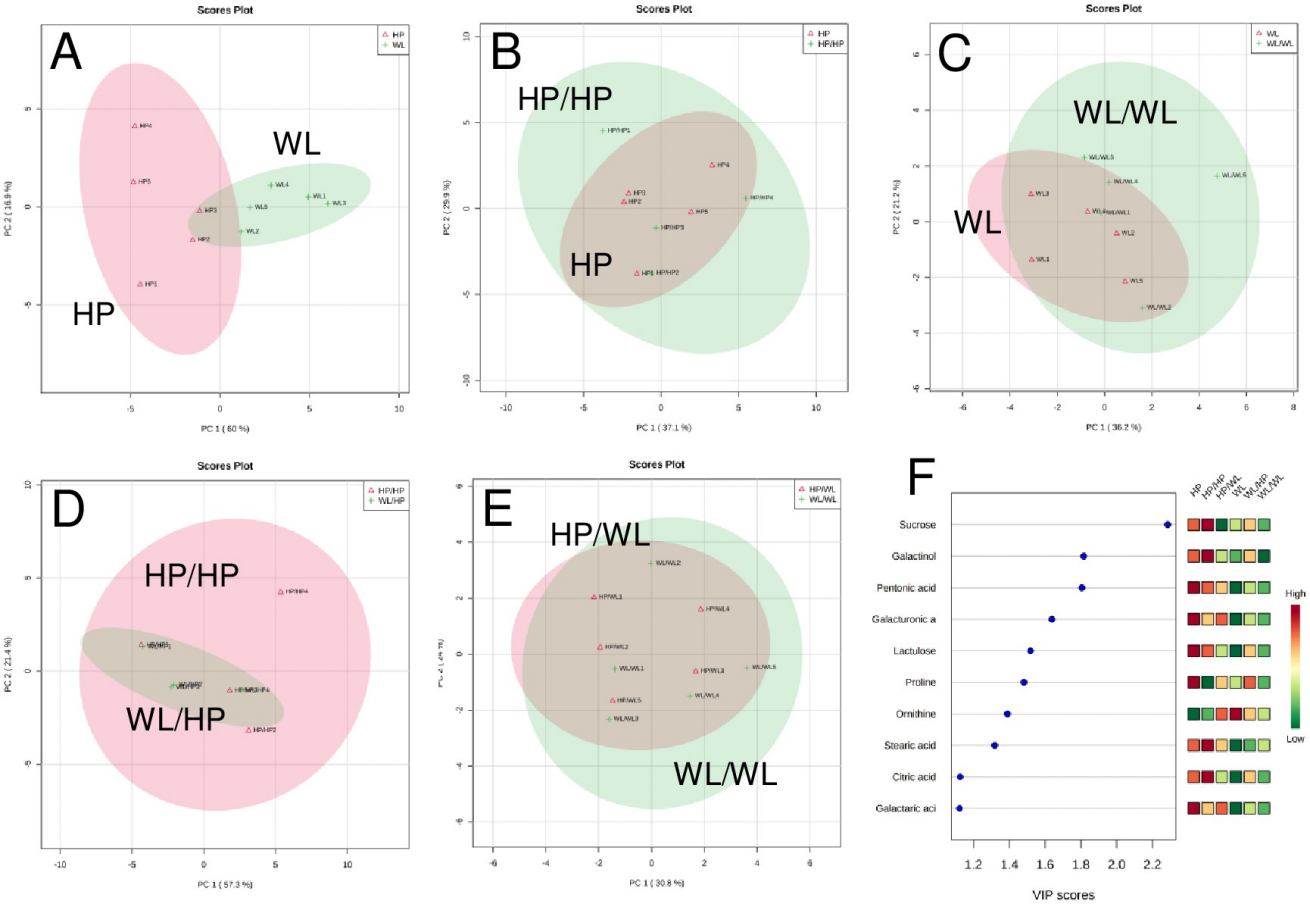
PCA and VIP plots from PLS-DA showing the metabolite differences in freshly harvested mature tubers of non-grafted (A), homo-grafted (B and C) and hetero-grafted (D and E) plants. The VIP plots (F) indicate the major differences between each category. In each plot, the 95% confidence regions are displayed. The tuber data were obtained from the same two consecutive experiments as those presented in Fig 3 for leaves. Each sample was prepared from 3 tubers approximately 1.5–2.0 cm in diameter. The data were obtained from 4–5 samples of each plant category. The labels are as in Fig 2.

The PCA plot showed that the metabolite compositions of the HP and WL tubers were quite different; however, they were not separated completely by the first and second components, which represented 57.3% and 21.4% of the total sample variance, respectively (Fig 4A). The major differences between the two cultivars were in the sugar, phenylalanine and ornithine contents (S7A Fig). The highest disparity was detected in the sucrose concentration, which was 47.3±11.0 in the HP tubers and 17.6±10.3 mg/g FW in the WP tubers (S4B Fig). Although the PCA biplots showed some differences (S7B–S7E Fig), the grafting, in general, did not severely change the metabolite profile of the tubers (Fig 4B–4E). Among the six different categories of tubers, a VIP score higher than 1.0 was found for 10 compounds, of which sucrose, galactinol, pentonic acid, galacturonic acid and lactulose had values higher than 1.5 (Fig 4F).

### Testing the effect of grafting on specialised metabolites

To gain insight into the effect of grafting on the concentrations of the metabolites highlighted by the VIP plots as the most important features separating the different plant categories (i.e., those compounds with values >1.5), bar graphs were prepared for each compound, and ANOVA with post hoc Tukey HSD tests were applied to identify the significant differences.

**Fig 5:**
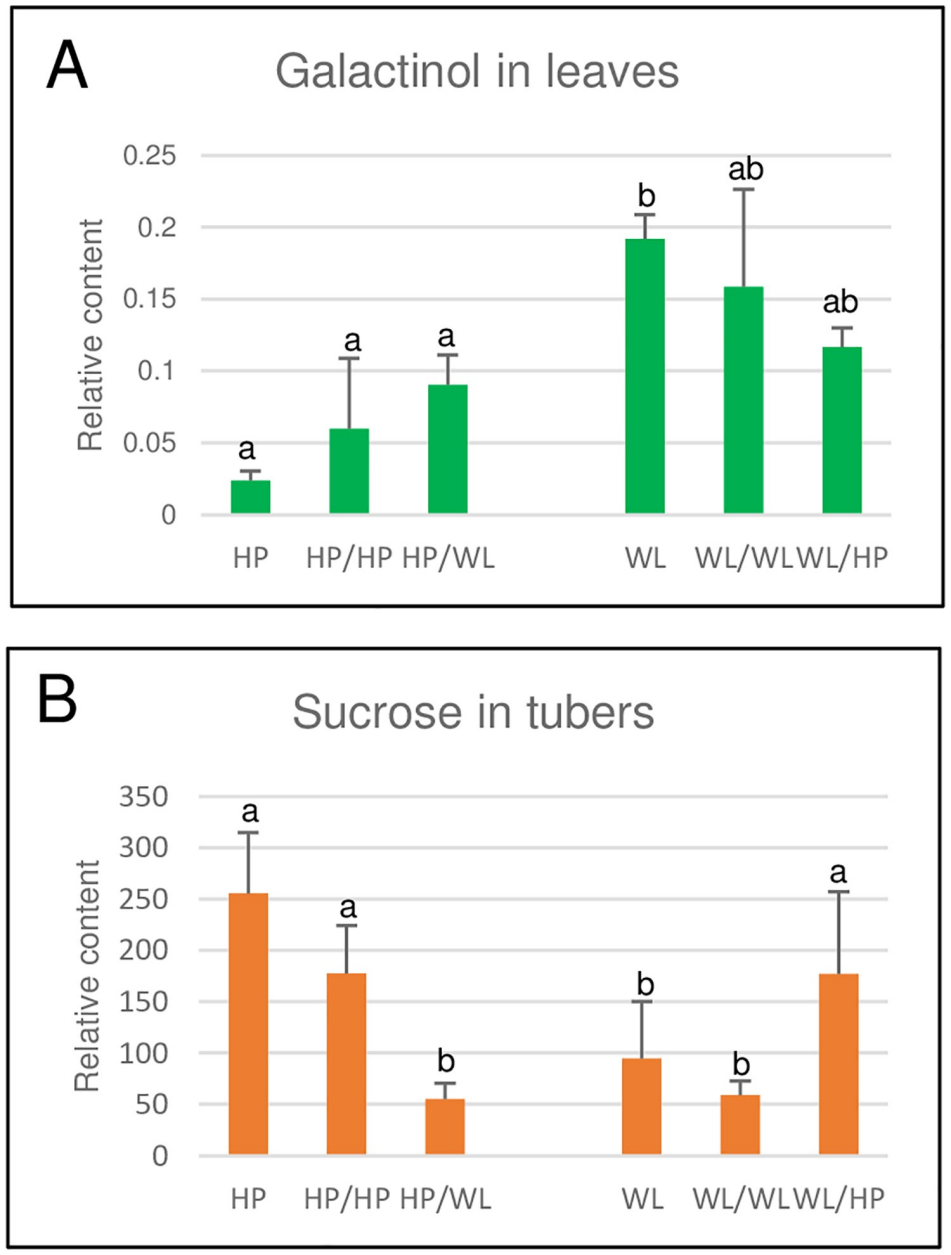
Differences in the galactinol and sucrose contents of leaves and tubers, respectively, detected by GC-MS; the relative data shown on the y axis are derived from a comparison of the peak sizes of the samples and an internal standard, ribitol. The means were obtained from the data presented in S1 and S2 Tables. The standard deviations are indicated by the error bars. The means presented with the same letter are not significantly different at *p* ≤ 0.05 calculated by one-way ANOVA with post hoc Tukey HSD test. The labels of the x axis are as in Fig 2.

In the leaves, four compounds had a value >1.5 (Fig 3F); however, only galactinol, the metabolite with the highest VIP score, showed a tendency of grafting-dependent changes. The galactinol concentration was 8-fold lower in the HP leaves than in the WL leaves. The grafting, especially hetero-grafting, increased the amount of galactinol in the HP leaves and decreased it in the WL leaves. However, none of these changes were significant at the *p* ˂ 0.05 level (Fig 5A).

In the case of the tubers, five compounds had a VIP score >1.5 (Fig 4F); of those compounds, as in the leaves, only the concentration of the most important compound, sucrose, was significantly (*p* ˂ 0.01–0.05) different between the tubers of the homo- and hetero-grafted plants. Fig 5B shows that there was a 2.7-fold difference between the sucrose content of the HP and WL tubers and a 3.0-fold difference between the sucrose content of the HP/HP and WL/WL tubers, and these differences were not changed significantly by hetero-grafting (Fig 5B). Thus, it was concluded that the sucrose concentration in tubers is genetically determined and is not substantially influenced by the signals derived from scions.

## Discussion

In the past, grafting experiments have been successfully used to identify signals of potato tuber development initiation [1, 17]. However, the mechanisms underlying the quality traits of tubers still have not been explored in detail. In this study, grafting experiments were carried out to unravel the influence of vegetative organs on the primary polar metabolite content of tubers.

Two potato cultivars, Hópehely (HP) and White Lady (WL), were homo- and hetero-grafted, and the effects of grafting were investigated in comparison to the non-grafted controls. In a previous study, Uri et al. [24] found a large difference in the metabolite composition of HP and WL tubers grown from first generation seed tubers under open air screen-house conditions. In this study, plants were propagated *in vitro*, potted and grown further under greenhouse conditions. Although the majority of the detected compounds were identical in the previous and current study, the PCA plots of the tubers grown in a screen-house had a higher separation rate than those grown in a greenhouse ([24] and Fig 4A). This difference might be explained by the better controlled environmental conditions in a greenhouse than in a screen-house. An alternative explanation may be based on the genetic diversity of the starting material, which could be different in the case of seed tubers and *in vitro* propagated plants.

The major difference between the HP and WL tubers was detected in the sucrose concentration, which was higher in the HP than in the WL tubers. These concentrations, however, were not influenced by hetero-grafts (Fig 5B). Sugar transport between source and sink tissues has been extensively studied, and it is mainly facilitated by the translocation of sucrose molecules [28]. Several experiments have shown that by manipulating the expression level of enzymes involved in starch synthesis, the sucrose and glucose contents of tubers can be strongly influenced [29–34]. In those experiments, however, the expression of target genes was manipulated by the expression of a suitable construct driven by the *CaMV35S*, *PATATIN* or *rolC* promoter, either in the whole plant or in a tuber- or vascular tissue-specific manner. In contrast, in our experiments, only natural signals derived from scions could influence gene expression and/or metabolite pathway regulation in rootstocks and vice versa. Since no significant alterations in the sucrose concentrations of the tubers developed on hetero-grafted compared to homo-grafted plants were detected, it was concluded that the sucrose content of the HP and WL tubers is under genetic control.

Differences between the metabolite profiles of the HP and WL plants were detected not only in the tubers but also in the leaves; the latter was mainly due to the 8-fold higher amount of galactinol in the leaves of the WL plants than in the leaves of the HP plants. Although no significant differences between the galactinol levels of the leaves of the homo- and hetero-grafted plants were found, there was a tendency for changes (Fig 5A), suggesting that the concentration of this compound is influenced by hetero-rootstocks. Galactinol is formed from UDP-galactose and myo-inositol. Galactosyl-sucrose oligosaccharides, such as raffinose and stachyose, are synthesised from galactinol and sucrose and from galactinol and raffinose, respectively, producing myo-inositol as a by-product [35]. The level of myo-inositol was similar in the leaves of all the plants (S1 Table), while the amounts of raffinose and stachyose were under the detection level. Thus, we suppose that the different galactinol concentrations in the different plant categories are related to differences in the rate of catabolism or phloem transport from source to sink organs rather than to galactinol or galactinol-derived oligosaccharide synthesis. Although it did not reach the level of significance, the amount of galactinol in the tubers was opposite of that detected in leaves, namely, it was higher in the HP than in the WL tubers and was largely unchanged by hetero-grafting (Fig 4F). Together, these data suggest that there is an intricate and yet unexplored molecular mechanism controlling galactinol metabolism and transport in the source-sink relation in potato.

Traits other than sucrose or the galactinol content of tubers can be influenced by grafting. Zhang and Guo [36] demonstrated that after grafting with tomato, the starch content and vitamin C level in potato tubers were significantly decreased but the reducing sugar content increased. The tuber yield decreased, and several tubers sprouted at harvest, which might be the reason for the reduced starch and elevated reducing sugar concentrations of the tubers. Tomato/potato hetero-grafting decreased the stolon number and stolon length, increased the gibberellic acid content of stolons and tubers and changed the expression of almost three thousand genes [37]. Furthermore, as presented in detail in the Introduction, grafting experiments have proven that the initiation of tuberisation is mediated by graft-transmissible signals. Our results are consistent with this finding, showing that the time of tuber initiation is delayed in WL rootstocks by HP scions and shortened in HP rootstocks by WL scions (Fig 2). In addition, we found a positive correlation between the growth rate of leaves and the earliness of tuberisation. In terms of lifetime, potato genotypes belong to three categories: early-, middle- and late-maturing genotypes. Studying an extensive set of genotypes representing different foliage maturity types under field conditions, a positive correlation between canopy growth parameters and tuber fresh weight was detected [38]. These field experiments, however, did not allow clear distinction between the effects on tuberisation and tuber bulking, while our experiment clearly showed a correlation between the rate of leaf growth and the time of tuber initiation. Nevertheless, further experiments involving a large number of genotypes are necessary to see how general this correlation is in different potato cultivars.

## Conclusions

We identified the major polar metabolites in leaves and tubers of two commercial potato cultivars and found characteristic differences in the metabolite compositions in both organs of the two cultivars. In comparison to non-grafted and homo-grafted controls no major effect of hetero-scions and rootstocks on the metabolite concentrations were detected suggesting that the level of major metabolites are under genetic control. The only exception was galactinol, the concentration of which was slightly influenced by hetero-grafting. Furthermore, the grafting experiments resulted in detection of a positive correlation between the rate of leaf growth and the time of tuber initiation in the case of the two tested cultivars.

## Supporting information

S1 Fig Morphology of mature tubers collected from the non-grafted and grafted potato plants. (PPTX)

S2 Fig Tuber number, tuber size and tuber yield of non-grafted and grafted potato plants. (PPTX)

S3 Fig Metabolite composition of leaves. (PPTX)

S4 Fig Sugar concentrations in the leaves and tubers. (PPTX)

S5 Fig PCA biplot analysis of the leaf metabolite data. (PPTX)

S6 Fig Metabolite composition of tubers. (PPTX)

S7 Fig PCA biplot analysis of the tuber metabolite data. (PPTX)

S1 Table Metabolites in the leaf extracts. (XLSX)

S2 Table Metabolites in the tuber extracts. (XLSX)
