# Supplementary material for: Metabolite analysis of tubers and leaves of two potato cultivars and their grafts
Source: PLoS One. 2021 May 6;16(5):e0250858. doi: 10.1371/journal.pone.0250858 (PMC8101760; doi:10.1371/journal.pone.0250858)
Supplement: S1 Fig — (PPTX) [file pone.0250858.s001.pptx]

## Slide 1
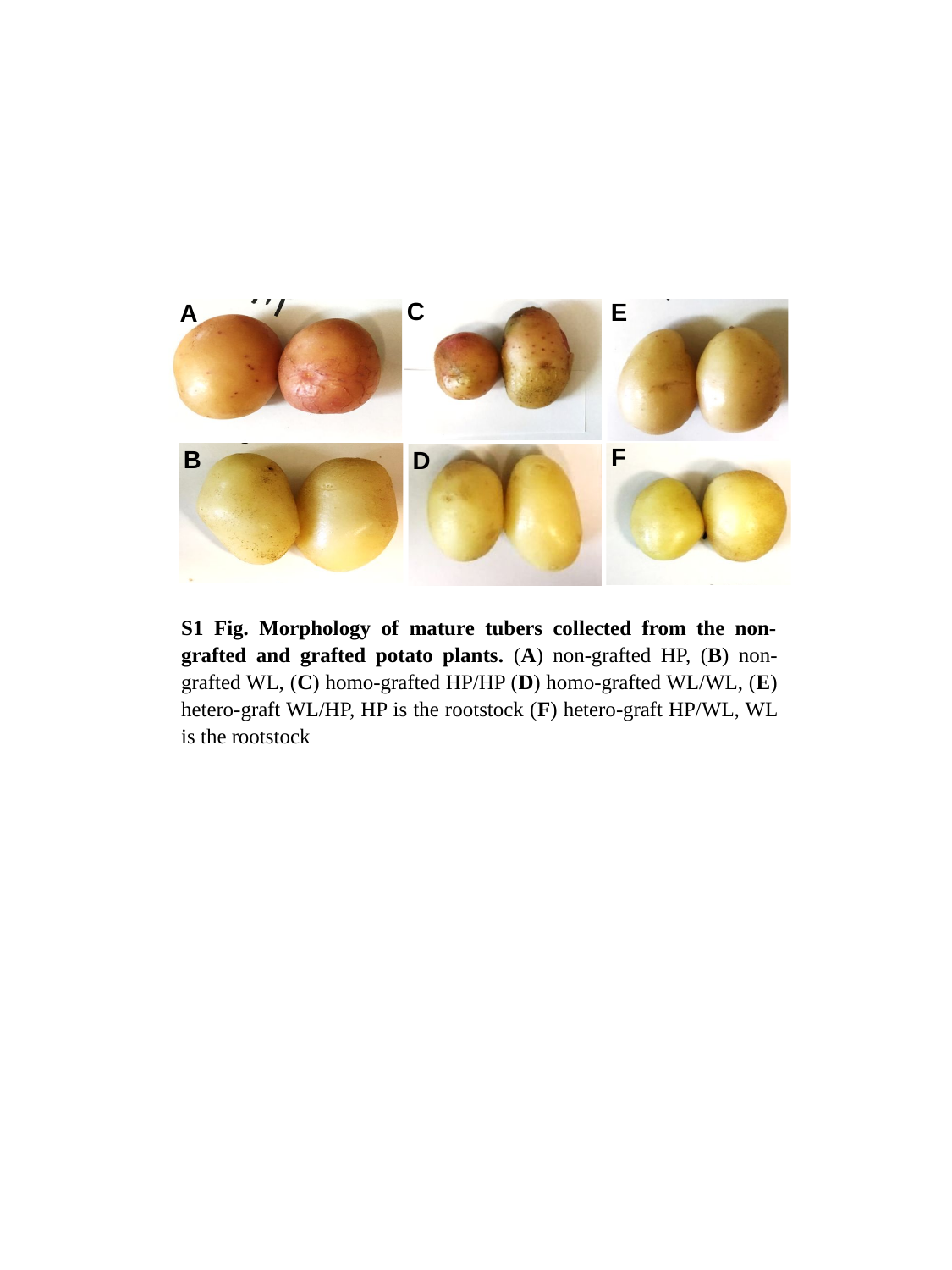

C
E
A
F
B
D
S1 Fig. Morphology of mature tubers collected from the non-grafted and grafted potato plants. (A) non-grafted HP, (B) non-grafted WL, (C) homo-grafted HP/HP (D) homo-grafted WL/WL, (E) hetero-graft WL/HP, HP is the rootstock (F) hetero-graft HP/WL, WL is the rootstock
