# Supplementary material for: Metabolite analysis of tubers and leaves of two potato cultivars and their grafts
Source: PLoS One. 2021 May 6;16(5):e0250858. doi: 10.1371/journal.pone.0250858 (PMC8101760; doi:10.1371/journal.pone.0250858)
Supplement: S2 Fig — (PPTX) [file pone.0250858.s002.pptx]

## Slide 1
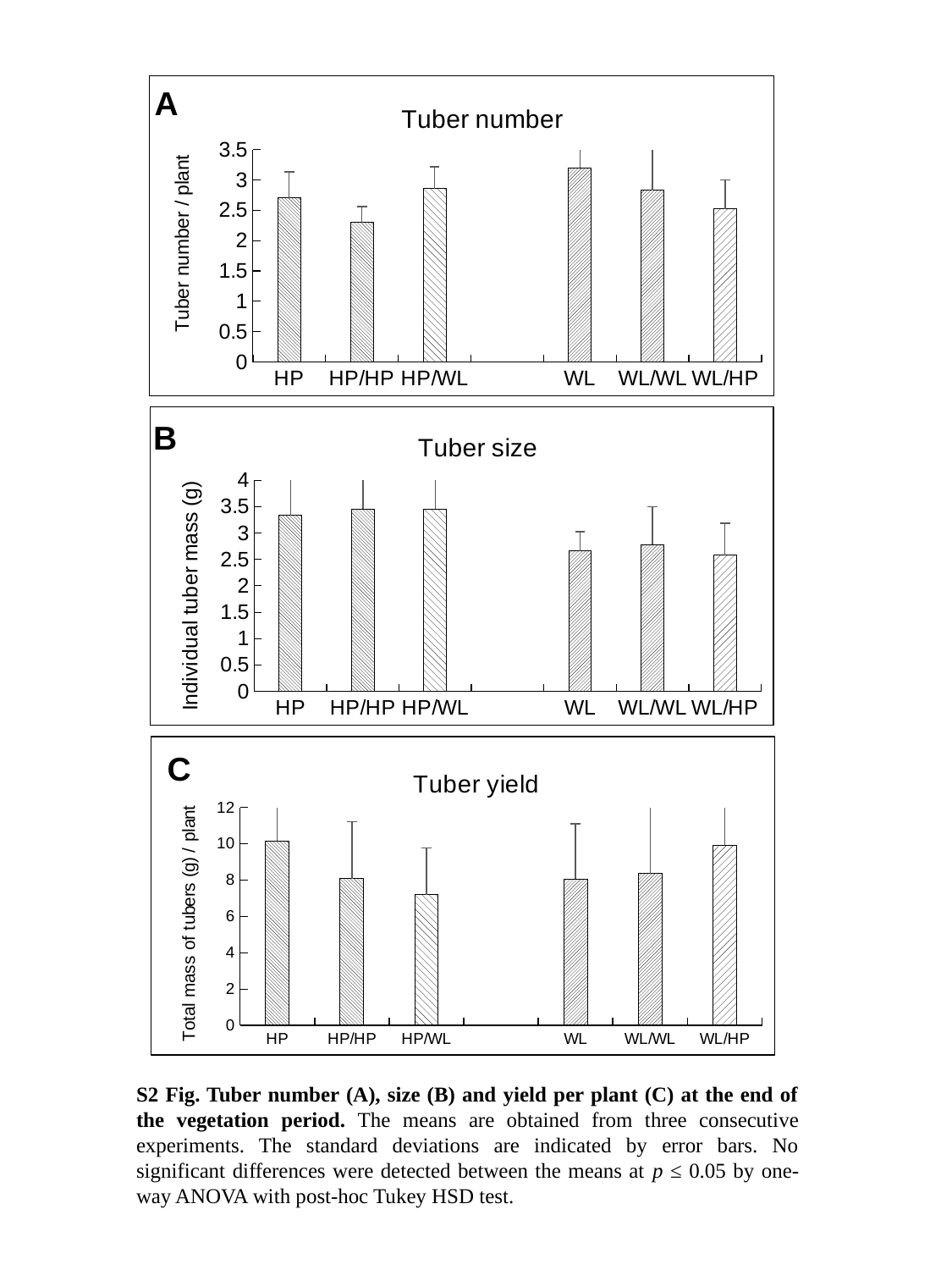

### Chart: Tuber number
| Category | |
|---|---|
| HP | 2.706666666666667 |
| HP/HP | 2.3000000000000003 |
| HP/WL | 2.86 |
| | None |
| WL | 3.1999999999999997 |
| WL/WL | 2.8333333333333335 |
| WL/HP | 2.523333333333335 |A
### Chart: Tuber size
| Category | |
|---|---|
| HP | 3.3299999999999987 |
| HP/HP | 3.456666666666663 |
| HP/WL | 3.453333333333333 |
| | None |
| WL | 2.66 |
| WL/WL | 2.781666666666667 |
| WL/HP | 2.5766666666666667 |B
### Chart: Tuber yield
| Category | |
|---|---|
| HP | 10.162714285714303 |
| HP/HP | 8.077111111111094 |
| HP/WL | 7.199166666666666 |
| | None |
| WL | 8.054666666666677 |
| WL/WL | 8.365555555555574 |
| WL/HP | 9.933094405594403 |C
S2 Fig. Tuber number (A), size (B) and yield per plant (C) at the end of the vegetation period. The means are obtained from three consecutive experiments. The standard deviations are indicated by error bars. No significant differences were detected between the means at p ≤ 0.05 by one-way ANOVA with post-hoc Tukey HSD test.
