# Supplementary material for: Metabolite analysis of tubers and leaves of two potato cultivars and their grafts
Source: PLoS One. 2021 May 6;16(5):e0250858. doi: 10.1371/journal.pone.0250858 (PMC8101760; doi:10.1371/journal.pone.0250858)
Supplement: S3 Fig — (PPTX) [file pone.0250858.s003.pptx]

## Slide 1
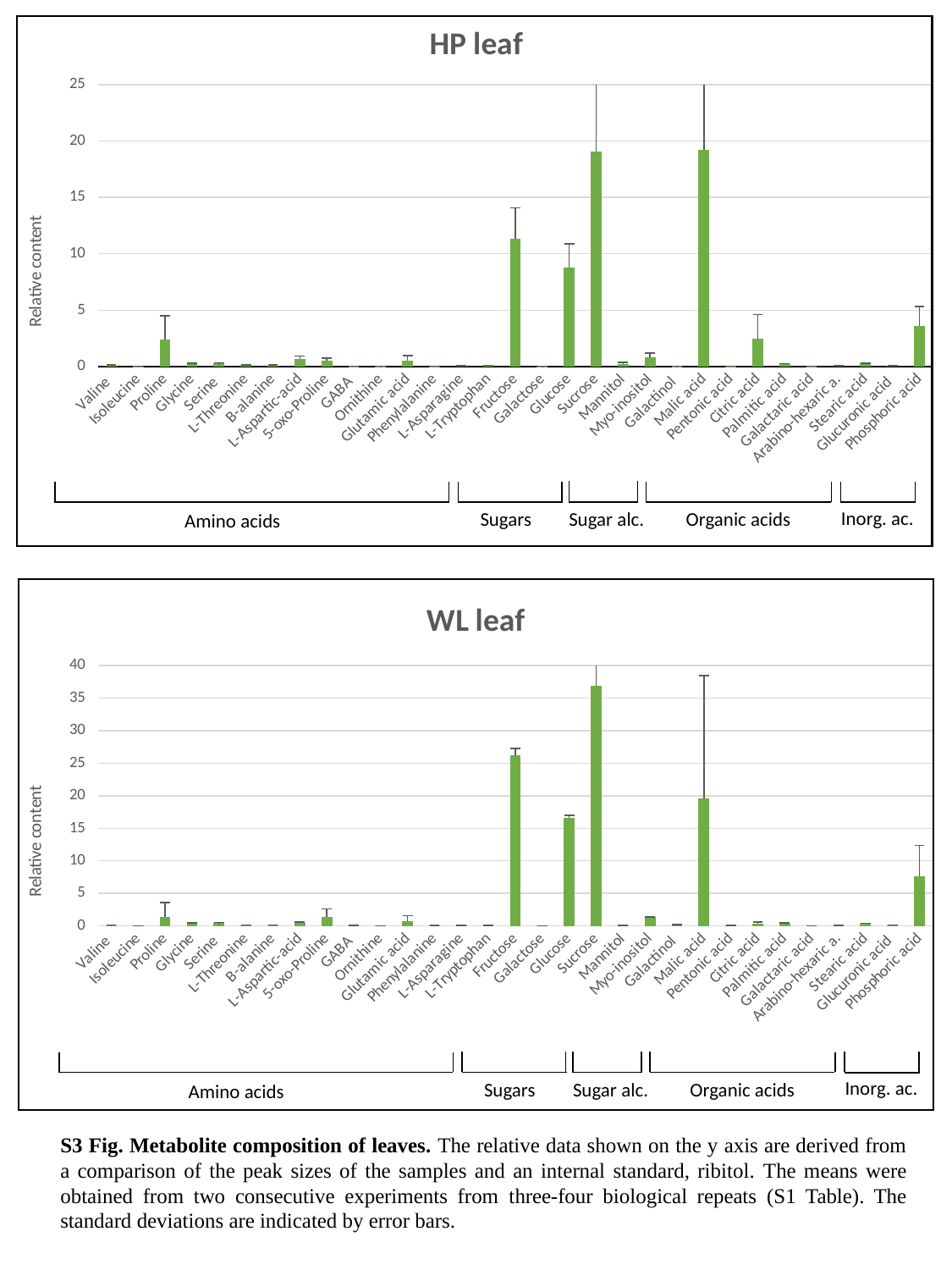

### Chart: HP leaf
| Category | |
|---|---|
| Valine | 0.08376326374068177 |
| Isoleucine | 0.017407006947618545 |
| Proline | 2.4232881905487207 |
| Glycine | 0.2410266345716562 |
| Serine | 0.315251954229802 |
| L-Threonine | 0.14669579845129924 |
| B-alanine | 0.07151370577191989 |
| L-Aspartic-acid | 0.6637654277696377 |
| 5-oxo-Proline | 0.5270655842800396 |
| GABA | 0.032350333845794006 |
| Ornithine | 0.014109516201296524 |
| Glutamic acid | 0.4992925357565108 |
| Phenylalanine | 0.03449784453101158 |
| L-Asparagine | 0.042639844863879686 |
| L-Tryptophan | 0.07613072673852002 |
| Fructose | 11.305100771570546 |
| Galactose | 0.02272891200000001 |
| Glucose | 8.77040310795132 |
| Sucrose | 19.06553389386436 |
| Mannitol | 0.22043300887285572 |
| Myo-inositol | 0.8061114035139566 |
| Galactinol | 0.023719199810217657 |
| Malic acid | 19.21290061929222 |
| Pentonic acid | 0.029354418432996517 |
| Citric acid | 2.4976539728011677 |
| Palmitic acid | 0.2587450341142818 |
| Galactaric acid | 0.028246029242000703 |
| Arabino-hexaric a. | 0.050454238287123816 |
| Stearic acid | 0.284421761186012 |
| Glucuronic acid | 0.0667939691185341 |
| Phosphoric acid | 3.6201903584874247 |
Inorg. ac.
Sugars
Sugar alc.
Organic acids
Amino acids
### Chart: WL leaf
| Category | |
|---|---|
| Valine | 0.08419940586844686 |
| Isoleucine | 0.022549849471061055 |
| Proline | 1.3779742808852715 |
| Glycine | 0.2936531266900082 |
| Serine | 0.4123088516794299 |
| L-Threonine | 0.1274424291208562 |
| B-alanine | 0.0714880494753852 |
| L-Aspartic-acid | 0.4509054861160016 |
| 5-oxo-Proline | 1.3663997644987684 |
| GABA | 0.051491697844662516 |
| Ornithine | 0.017791545805430343 |
| Glutamic acid | 0.7400087618608675 |
| Phenylalanine | 0.052934687838232075 |
| L-Asparagine | 0.03529426101770601 |
| L-Tryptophan | 0.06141577903664942 |
| Fructose | 26.248181042585085 |
| Galactose | 0.02791044233333334 |
| Glucose | 16.59042516320899 |
| Sucrose | 36.878304476868074 |
| Mannitol | 0.03544610179014667 |
| Myo-inositol | 1.3286049320644358 |
| Galactinol | 0.19189550791704937 |
| Malic acid | 19.585059378736094 |
| Pentonic acid | 0.051448442472523656 |
| Citric acid | 0.32478976945904453 |
| Palmitic acid | 0.30612442472208024 |
| Galactaric acid | 0.022423340069397512 |
| Arabino-hexaric a. | 0.07454373865601732 |
| Stearic acid | 0.28290099405026053 |
| Glucuronic acid | 0.09281376715824735 |
| Phosphoric acid | 7.648356422724672 |Inorg. ac.
Sugars
Sugar alc.
Organic acids
Amino acids
S3 Fig. Metabolite composition of leaves. The relative data shown on the y axis are derived from a comparison of the peak sizes of the samples and an internal standard, ribitol. The means were obtained from two consecutive experiments from three-four biological repeats (S1 Table). The standard deviations are indicated by error bars.
