# Supplementary material for: Metabolite analysis of tubers and leaves of two potato cultivars and their grafts
Source: PLoS One. 2021 May 6;16(5):e0250858. doi: 10.1371/journal.pone.0250858 (PMC8101760; doi:10.1371/journal.pone.0250858)
Supplement: S4 Fig — (PPTX) [file pone.0250858.s004.pptx]

## Slide 1
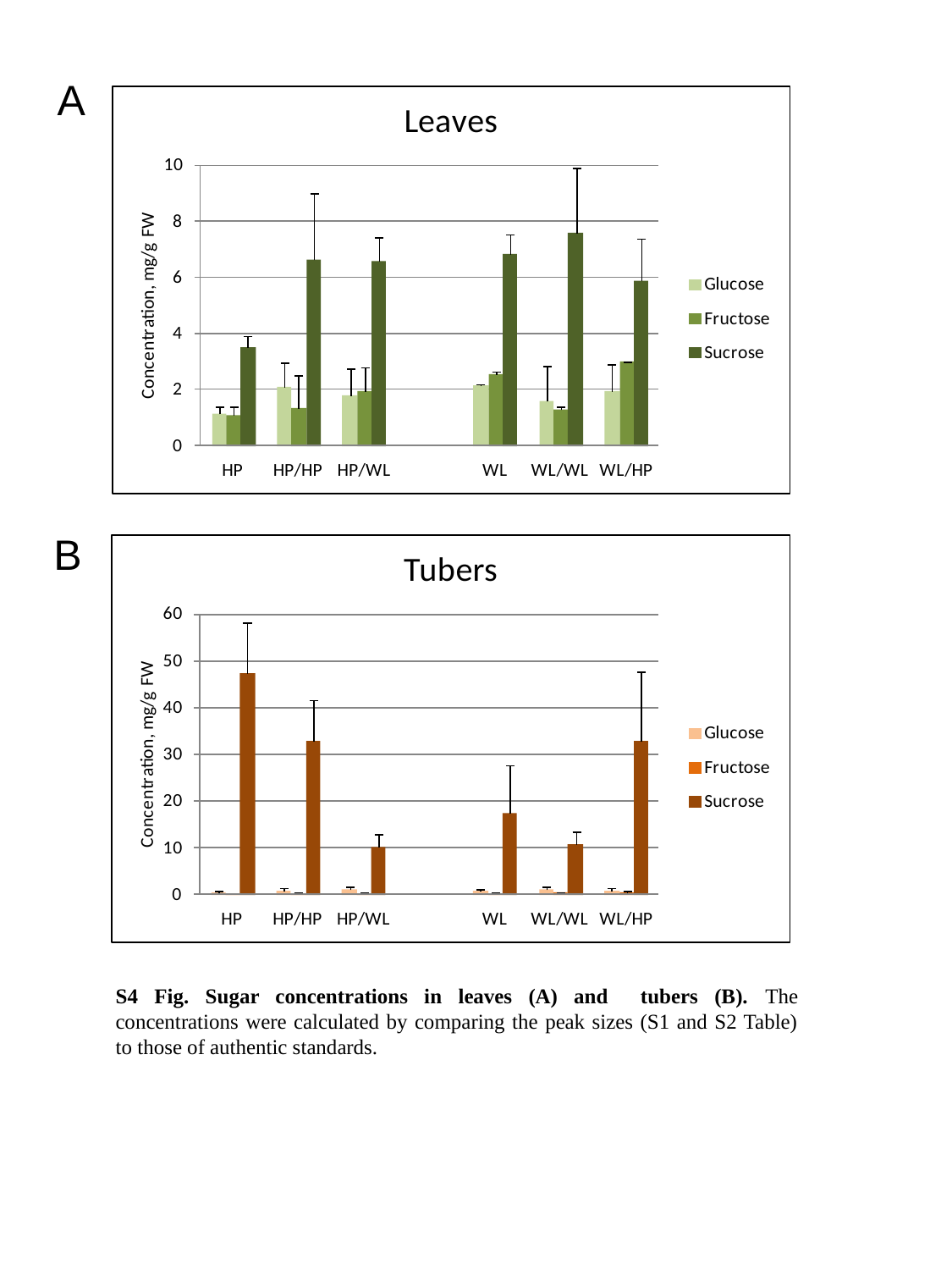

A
B
S4 Fig. Sugar concentrations in leaves (A) and tubers (B). The concentrations were calculated by comparing the peak sizes (S1 and S2 Table) to those of authentic standards.
