# Supplementary material for: Metabolite analysis of tubers and leaves of two potato cultivars and their grafts
Source: PLoS One. 2021 May 6;16(5):e0250858. doi: 10.1371/journal.pone.0250858 (PMC8101760; doi:10.1371/journal.pone.0250858)
Supplement: S5 Fig — (PPTX) [file pone.0250858.s005.pptx]

## Slide 1
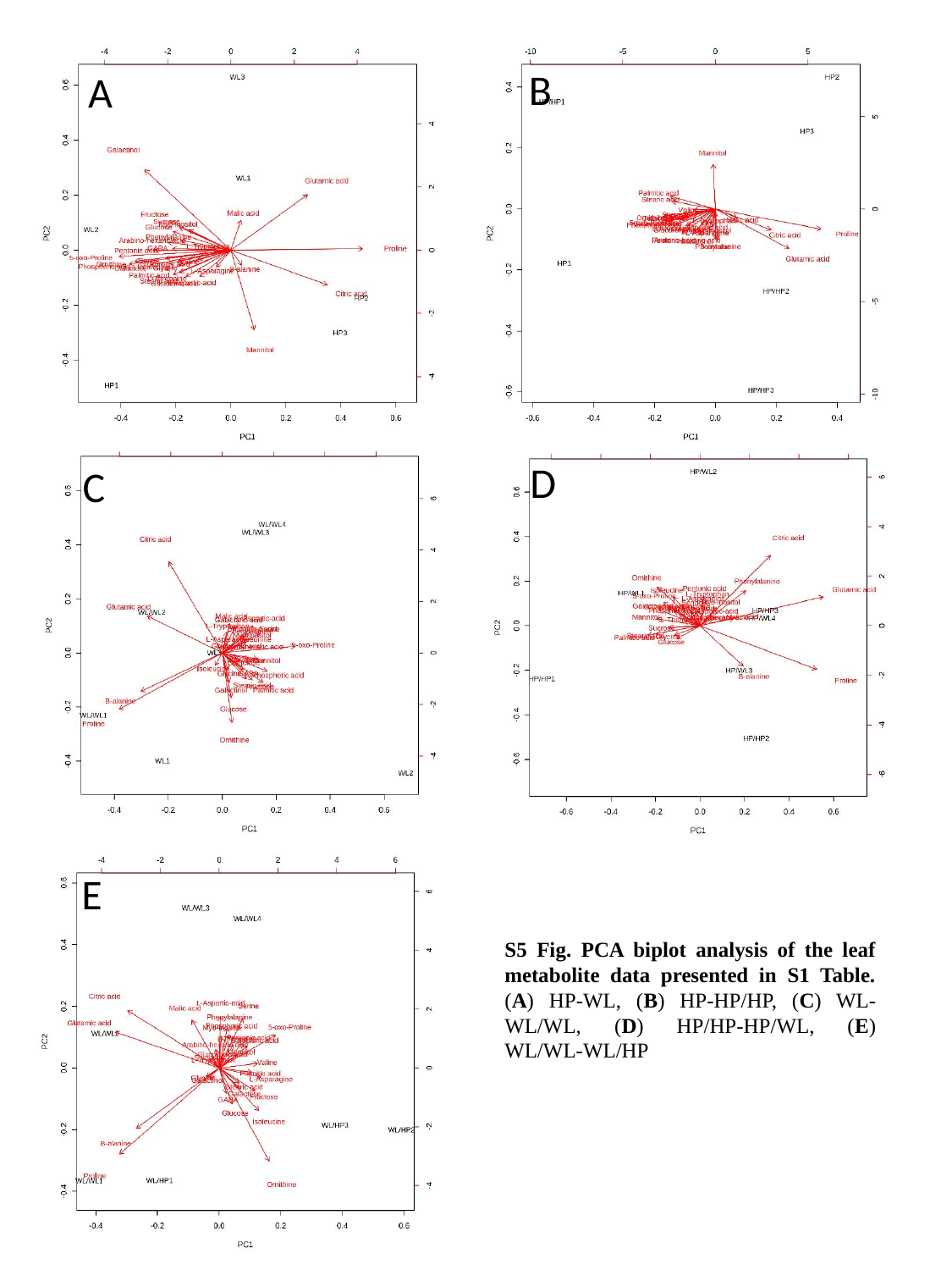

B
A
D
C
E
S5 Fig. PCA biplot analysis of the leaf metabolite data presented in S1 Table. (A) HP-WL, (B) HP-HP/HP, (C) WL-WL/WL, (D) HP/HP-HP/WL, (E) WL/WL-WL/HP
