# Supplementary material for: Metabolite analysis of tubers and leaves of two potato cultivars and their grafts
Source: PLoS One. 2021 May 6;16(5):e0250858. doi: 10.1371/journal.pone.0250858 (PMC8101760; doi:10.1371/journal.pone.0250858)
Supplement: S6 Fig — (PPTX) [file pone.0250858.s006.pptx]

## Slide 1
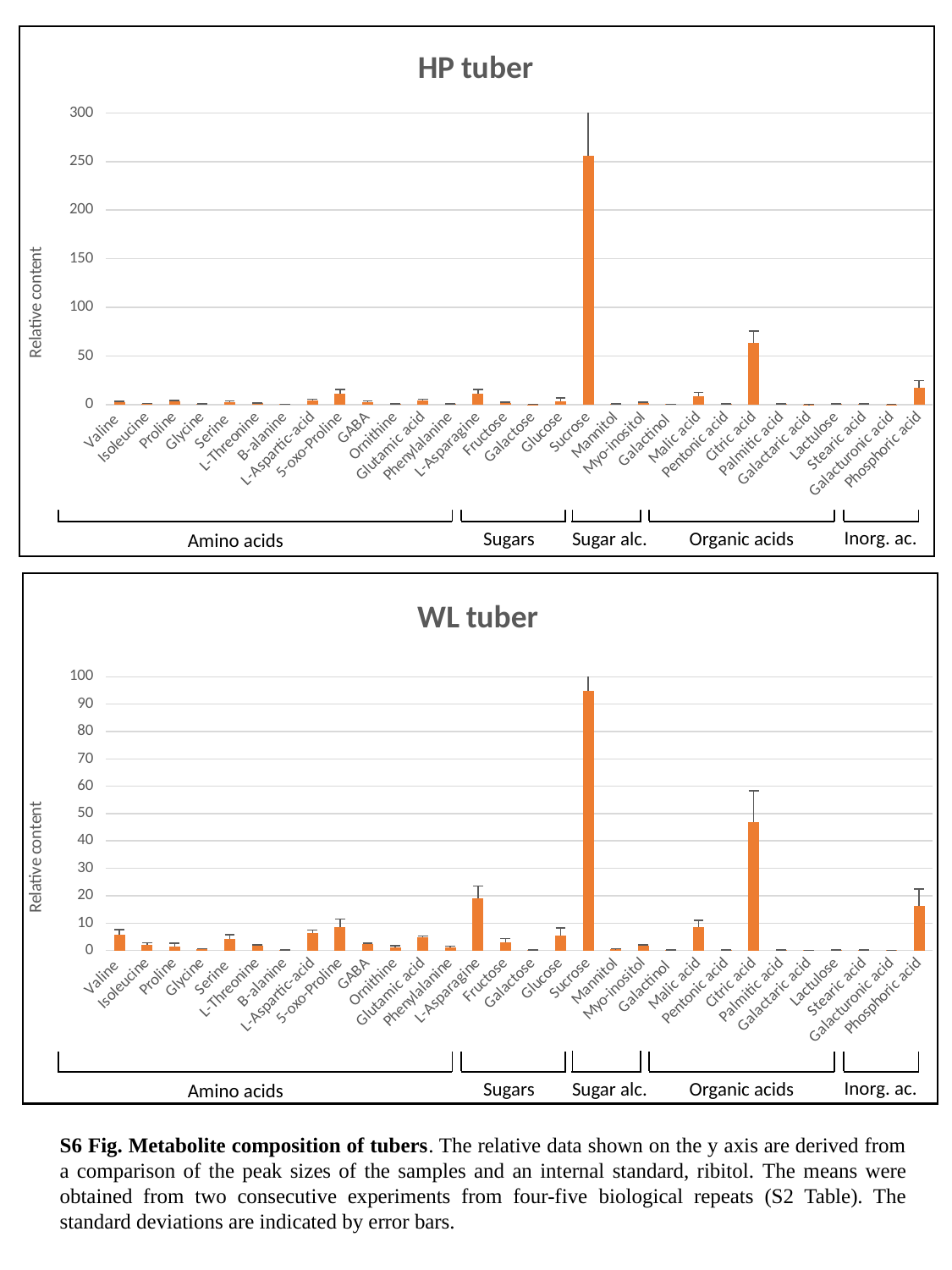

### Chart: HP tuber
| Category | |
|---|---|
| Valine | 2.613055607502706 |
| Isoleucine | 0.9015193923975018 |
| Proline | 3.447616018100974 |
| Glycine | 0.58351370582096 |
| Serine | 2.365593444724255 |
| L-Threonine | 1.1469943174495802 |
| B-alanine | 0.2525857485494748 |
| L-Aspartic-acid | 4.366696867781932 |
| 5-oxo-Proline | 11.099917985375667 |
| GABA | 2.532961066166169 |
| Ornithine | 0.3183241943737931 |
| Glutamic acid | 3.8814585119758056 |
| Phenylalanine | 0.3167579053619913 |
| L-Asparagine | 11.510883055224674 |
| Fructose | 1.6547790007964593 |
| Galactose | 0.20270612800000004 |
| Glucose | 3.5950281295876167 |
| Sucrose | 255.53788203127388 |
| Mannitol | 0.5258235322553397 |
| Myo-inositol | 1.892137225692448 |
| Galactinol | 0.21957435441812045 |
| Malic acid | 8.778211777400958 |
| Pentonic acid | 0.3129133765260783 |
| Citric acid | 63.109223258513424 |
| Palmitic acid | 0.33725714458191525 |
| Galactaric acid | 0.08924186972995472 |
| Lactulose | 0.35043456321914296 |
| Stearic acid | 0.4295359684783924 |
| Galacturonic acid | 0.11471699727355562 |
| Phosphoric acid | 17.611977925541602 |Inorg. ac.
Sugars
Sugar alc.
Organic acids
Amino acids
### Chart: WL tuber
| Category | |
|---|---|
| Valine | 5.8954986324750145 |
| Isoleucine | 2.004870758973004 |
| Proline | 1.5753426031901228 |
| Glycine | 0.6114665129285527 |
| Serine | 4.187641304463532 |
| L-Threonine | 1.7744592523043803 |
| B-alanine | 0.2108804770193294 |
| L-Aspartic-acid | 6.38379328618394 |
| 5-oxo-Proline | 8.461111994840099 |
| GABA | 2.23636614830108 |
| Ornithine | 1.0405181294194081 |
| Glutamic acid | 4.789931118098521 |
| Phenylalanine | 1.0706293351702778 |
| L-Asparagine | 18.959058492834107 |
| Fructose | 2.843997747675802 |
| Galactose | 0.1676456748 |
| Glucose | 5.538110775615776 |
| Sucrose | 94.81684306702485 |
| Mannitol | 0.46465374809660026 |
| Myo-inositol | 1.7992826733344238 |
| Galactinol | 0.14196356823118297 |
| Malic acid | 8.6136353656267 |
| Pentonic acid | 0.1401946849912374 |
| Citric acid | 46.998663700929946 |
| Palmitic acid | 0.19244658645695228 |
| Galactaric acid | 0.058485508820882506 |
| Lactulose | 0.1669565344295329 |
| Stearic acid | 0.1924971853735414 |
| Galacturonic acid | 0.0570292111058212 |
| Phosphoric acid | 16.126720385040432 |Inorg. ac.
Sugars
Sugar alc.
Organic acids
Amino acids
S6 Fig. Metabolite composition of tubers. The relative data shown on the y axis are derived from a comparison of the peak sizes of the samples and an internal standard, ribitol. The means were obtained from two consecutive experiments from four-five biological repeats (S2 Table). The standard deviations are indicated by error bars.
